# Remedies and Their Uses

**Published:** 1907-05-04

**Authors:** 


### Remedies and their Uses.

### New Laxatives for Chronic Constipation.

*Regulin.*—Among the newer remedies for chronic constipation may be noted the interesting account by Voit (M. M. W., July 1906) of the employment of agar-agar, the well-known nutrient medium of the bacteriologist. Like other forms of gelatine it has the power of absorbing considerable amounts of water; it forms a somewhat heavy soft heap in the bowel, and thus acts mechanically as an intestinal stimulant. A little cascara freed from its bitter principle is added in the form of a 20 per cent. watery extract. The preparation is known as "regulin." At first one tablespoonful may be given daily, and if this is found, after a day or two, to be insufficient, the dose may be doubled. Obstinate cases at first will require an enema. When a regular action has been attained the dose may be reduced to a teaspoonful. No ill effects have been observed.

*Psyllium.*—The seeds of psyllium plantago have recently been carefully examined as to their chemico-physical properties by Dr. Langlebert, and his results are recorded in the "Journal de Médécine de Paris," 1906. Langlebert therefore concludes that psyllium, both from its mechanical action and from the facility with which the oils are liberated, will be active as an intestinal stimulant. The dose is one heaped teaspoonful moistened with a little water, and taken twice daily with food; or a tablespoonful may be taken at bedtime. Psyllium seeds may be obtained from Roberts and Co., New Bond Street, London.

*Anusol* is iodo-resorcin-sulphonate of bismuth, and is supplied only in the form of suppositories. They are said to have a non-irritant laxative action, and are recommended in chronic constipation, hæmorrhoids, etc. Besides the anusol the suppositories contain oxide of zinc and balsam of Peru. Ordinarily one suppository every night will be found efficacious, but occasionally one night and morning is required, and the treatment should be continued for some weeks, until the improvement is permanent. Dietetic and general measures should not meanwhile be neglected, as the medicinal treatment is only satisfactory as an adjuvant to rational general therapeutics. Anusol is prepared by Reitmeyer and Co.

